# Alterations of Oral Microbiota in Chinese Patients With Esophageal Cancer

**DOI:** 10.3389/fcimb.2020.541144

**Published:** 2020-10-21

**Authors:** Qiaofei Zhao, Tian Yang, Yifan Yan, Yu Zhang, Zhibin Li, Youchun Wang, Jing Yang, Yanli Xia, Hongli Xiao, Hongfeng Han, Chunfen Zhang, Weihong Xue, Hongyi Zhao, Hongwei Chen, Baoyong Wang

**Affiliations:** Department of Gastroenterology, Luoyang Central Hospital Affiliated to Zhengzhou University, Luoyang, China

**Keywords:** esophageal cancer, oral microbiota, 16S rDNA gene, alteration, indicator

## Abstract

Emerging evidence supports that oral microbiota are associated with health and diseases of the esophagus. How oral microbiota change in Chinese patients with esophageal cancer (EC) is unknown, neither is their biomarker role. For an objective to understand alterations of oral microbiota in Chinese EC patients, we conducted a case-control study including saliva samples from 39 EC patients and 51 healthy volunteers. 16S rDNA genes of V3-V4 variable regions were sequenced to identify taxon. Relationship between oral flora and disease was analyzed according to alpha diversity and beta diversity. Resultantly, the Shannon index (*p* = 0.2) and the Simpson diversity index (*p* = 0.071) were not significant between the two groups. Yet we still found several species different in abundance between the two groups. For the EC group, the most significantly increased taxa were *Firmicutes, Negativicutes, Selenomonadales, Prevotellaceae, Prevotella*, and *Veillonellaceae*, while the most significantly decreased taxa were *Proteobacteria, Betaproteobacteria, Neisseriales, Neisseriaceae*, and *Neisseria*. In conclusion, there are significant alterations in abundance of some oral microbiomes between the EC patients and the healthy controls in the studied Chinese participants, which may be meaningful for predicting the development of EC, and the potential roles of these species in EC development deserve further studies.

## Introduction

Esophageal cancer (EC) ranks the eighth in the most common cancers among the world (Bollschweiler et al., 2017; Short et al., 2017; Huang and Yu, 2018). Asia and Africa have the majority of patients with EC. The most commonly seen in Asia and Africa was esophageal squamous cell cancer, while in North America and Europe, the major type is esophageal adenocarcinoma (EAC) (Short et al., 2017; Huang and Yu, 2018). Both morbidity and mortality of EC remain considerably high worldwide. The difficulty in prevention and the shortage in specific biomarkers make the disease often found in advanced stages. Though magnifying endoscopy with narrow band imaging is considerably effective in detecting early esophageal squamous cell cancer and precancerous lesions, it is not sufficiently accurate in detecting early adenocarcinoma. Besides, the majority of patients in China have no access to this advanced detection, while the white light imaging is obviously less effective in detecting lesions. Furthermore, EC ranks fourth among malignant tumors in China and is a heavy burden of the health care system (Liu et al., 2015; Lin et al., 2017). Therefore, non-invasive, simple, more effective, and more specific methods to detect EC at an early stage, at which EC could achieve an *en bloc* dissection by endoscopic or surgical operation, are urgently needed.

Variations of diet habits, populations, regions, and age may play important roles in the occurrence and development of EC (Bollschweiler et al., 2017). So does the oral microbiota. Emerging evidence suggests that human microorganism is closely related to diseases in the digestive system (Bollschweiler et al., 2017; Peters et al., 2017; Short et al., 2017; Flemer et al., 2018; Huang and Yu, 2018; Xian et al., 2018; Xun et al., 2018; Graves et al., 2019). Oral microbiome ranks only the second in diversity and abundance to that of the gut. The esophagus is so close to the mouth that we propose that the oral microbiota may be related to esophageal diseases, such as EC and reflux esophagitis. Deshpande et al. (2018) conducted the most comprehensive assessment of the esophageal microbiome, finding that bacterial signatures and functions were closely related with the early stages of the EC cascade, such as enrichment with Gram-negative oral-associated bacteria (Deshpande et al., 2018). Existing studies prove that changes in the composition and function of the oral microbiota are involved in esophageal diseases like reflux esophagitis, Barrett's esophagus (BE), and EC (Cao, 2017; Ajayi et al., 2018; Corning et al., 2018; May and Abrams, 2018). Given that saliva samples are easy to collect, and high-throughput next-generation sequencing is widely used in detecting, identifying, and classifying microorganism (Sanschagrin and Yergeau, 2014; Xun et al., 2018), we conducted a case-control study to analyze the differences of oral microbiota in the composition of EC patients, intending to help developing new, non-invasive, and effective detections for early EC.

## Patients and Methods

### Patients

We recruited 39 EC patients, all from outpatients or inpatients in our hospital. All patients were diagnosed with EC by endoscopic detection and pathology for the first time. Histopathological types were identified by experienced pathologists through biopsy specimens or surgical specimens. Radiography examinations were done for every patient to evaluate clinical staging. We also enrolled 51 healthy volunteers matching the EC patients in age and sex to a certain extent. The healthy controls were unrelated patients' family members, doctors, and nurses in our hospital. All the volunteers were recruited from September 2018 to January 2019. General information is shown in Table 1.

**Table 1:** Clinical characteristics of EC patients and healthy controls.

**Characteristics**	**EC patients**	**Healthy controls**	***p*-values**
**Gender**			0.1919
Female	16	28	
Male	23	23	
**Age, years, mean (SD)**	60.39 (10.31)	49.18 (11.87)	0.0000^**^
**Smoking**			0.7452
No smoking	28	35	
Smoking	11	16	
**Alcohol drinking**			0.2565
No or little drinking	31	45	
Regular or heavy drinking	8	6	
**Fiber intake**			0.9206
Low fiber intake	21	28	
High fiber intake	18	23	
**Sugar intake**			0.0376^*^
Low sugar intake	31	30	
High sugar intake	8	21	
**Salt intake**			0.6760
Low salt intake	22	31	
High salt intake	17	20	
**Fat intake**			0.0575
Low fat intake	31	31	
High fat intake	8	20	
**Starch intake**			0.6143
Low starch intake	11	12	
High starch intake	28	39	
**Meat intake**			0.0544
Low meat intake	27	25	
High meat intake	12	26	
**Vegetables/fruits intake**			0.1919
Low vegetable/fruit intake	16	28	
High vegetable/fruit intake	23	23	

*EC, esophageal cancer; SD, standard deviation*.

* *p < 0.05*;

** *p < 0.001*.

In this study, patients who received any antineoplastic treatment or upper gastrointestinal surgery were excluded. Participants who received immunosuppressors or antibiotics longer than 4 weeks in the recent 6 months were eliminated. Volunteers older than 80 years, complicated with complex esophageal diseases (e.g., esophageal ulcers, eosinophilic esophagitis) or autoimmune diseases (e.g., rheumatoid arthritis, Sjogren's syndrome) were ruled out.

Questionnaire survey about past medical history, diet habits (e.g., vegetable/fruit intake, meat intake, salt intake, starch intake, sugar intake, fat intake), smoking, and alcohol drinking was done for every participant. Each subject signed a written informed consent for the scientific use of his/her saliva sample and clinical data. And we were committed not to reveal out volunteers' data. Our study was approved by the ethics committees of Luoyang Central Hospital affiliated to Zhengzhou University.

### Samples Collection

Participants were asked to rinse their mouths with warm water to eliminate food debris and then waited for 5 min or longer for natural saliva secretion, not speaking or taking in food or water. Each saliva sample was spit into a sterile container and not <3 ml. Every sample was then transferred into another sterile cryopreservation tube, using a sterile syringe, and stored into a −80°C freezer immediately until extracted for DNA.

### DNA Extraction and Sequencing

Total DNA was extracted from saliva samples using a PowerSoil DNA isolation kit (Qiagen, Valencia, CA, USA), in accordance with the manufacturer's instructions. PCR was then performed following a universal primer for 16s rDNA 341F/806R (341F: 5′-CCTACGGGRSGCAGCAG-3′; 806R: 5′-GGACTACVVGGGTATCTAATC-3′). V3-V4 variable region of each 16s rDNA was amplified *via* a KAPA HiFi Hotstart ReadyMix PCR kit. The PCR products were detected by 2% agarose gel electrophoresis and recovered by gel cutting with AxyPrep DNA gel Recovery Kit (AXYGEN company). NanoDrop 2000 spectrophotometer (Thermo Fisher Scientific, Waltham, MA, USA) and 2% agarose gel electrophoresis were then applied for quality control of the library. After that, the amplified genes were sequenced in Illumina Miseq PE250 instrument (Illumina, San Diego, CA, USA) with 2 × 250 base pair (bp) paired-end (PE) sequencing. All the DNA extraction, amplification, and sequencing were performed at Realbio Technology (Shanghai, China).

### Data Processing

PANDAseq (Masella et al., 2012) was applied to assemble overlapping PE reads that were overlapped or had a mean sequence quality <20 or had more than three bases containing N. The length of reads was controlled in 220–500 nt. Clean reads were then obtained for the analysis of downstream species diversity.

Long reads were clustered into operational taxonomic units (OTUs). Firstly, the singletons (corresponding to only one sequence of reads) in the spliced long reads are filtered out. USEARCH is then used to cluster reads into the same OTU when their distance-based similarity was >0.97 (Edgar, 2013). After chimeric filtering of clustered sequences, OTUs for species classification were obtained. It is considered that each OTU can represent a species.

**Figure 1:**
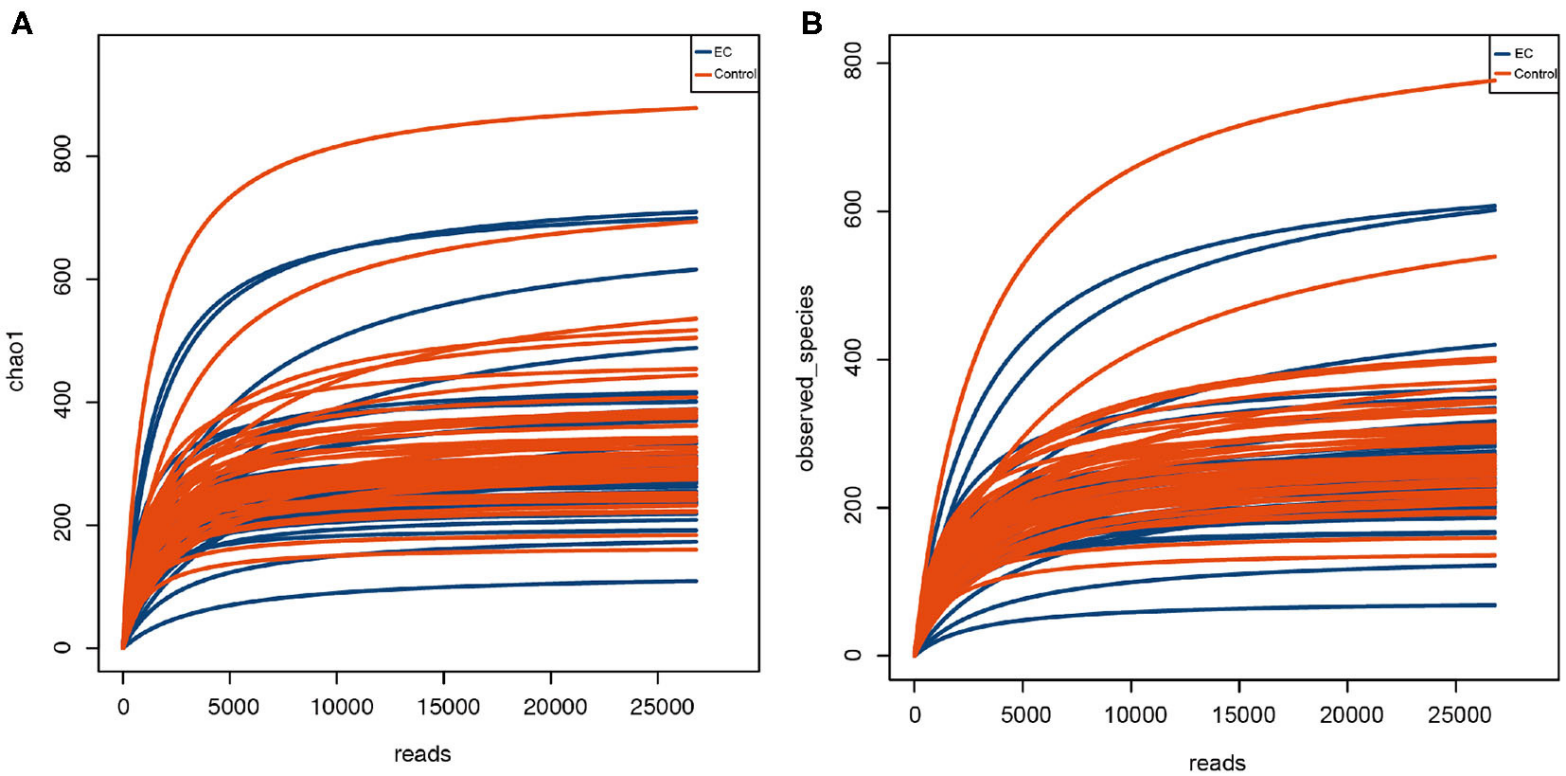
Rarefaction curve of alpha diversity index indicating species richness of oral microbiota. The abscissa represents the number of Clean Reads randomly selected from a sample, and the ordinate represents the species diversity within a single sample. The curve in the figure represents a sample. The curve tends to be flat as the depth of sequencing increases, indicating that the volume of sequencing data is reasonable. **(A)** Chao 1 index represents the total number of operational taxonomic units (OTUs) in the sample. **(B)** Observed species index represents the number of OTUs actually observed.

We randomly extracted the average value of each sample according to the minimum number of sequences matched to OTU when the samples reached sufficient sequencing depth (Figure 1). It was done to avoid the analysis deviation caused by different data sizes of samples. We extracted a read as a representative sequence from every OTU and compared them with the RDP database (RDP, http://rdp.cme.msu.edu; Wang et al., 2007; Cole et al., 2014). Each OTU was classified to a different species based on the comparison results. Species abundance tables were together obtained for the subsequent analysis.

### Statistics Analysis

Clinical data were analyzed by SPSS (ver. 25.0, SPSS Inc., Chicago, IL, USA). Student's *T*-test was used for quantitative variables, while Pearson's chi-square test was applied for categorical variables. Sequencing data were analyzed by R software (ver. 3.1.0, the R Project for Statistical Computing). A *p* < 0.05 was identified as statistically significant.

Alpha diversity is the analysis of species diversity in a single sample, including Observed species index, Chao1 index, Shannon index, and Simpson index. QIIME software was applied to calculate the value of alpha diversity index and to generate the corresponding rarefaction curves. Alpha diversity indices with significant differences under different conditions were screened by rank sum test (using Wilcoxon test function in R).

Beta diversity analysis was used to compare differences in species diversity among samples. Phylogenetic distances of the system were calculated by weighted UniFrac to compare species community differences among samples, which took the evolutionary distance between species into account. A larger index means a greater difference between samples. Based on the weighted UniFrac analysis, principal coordinate analysis (PCoA) was used to show differences between the samples. The results indicated that if the two samples were close, the species compositions of the two samples were similar. Heatmap clustered samples with similar beta diversity, reflecting similarities between samples. One-way analysis of similarities (ANOSIM) was used to test the significance of the difference between the two groups. Linear discriminant analysis (LDA) effect size (LEfSe) was used to estimate the impact of abundance of each species on the difference effect and to find out the communities or species in different groups that had an impact on the significance difference. LDA threshold was 2. Rank sum test (Wilcoxon test function of R language stats package) was used to analyze the significant differences among different groups in order to find out the species that had significant differences in the division of groups. A *p* < 0.05 was identified as statistically significant. All *p*-values were adjusted by false discovery rate (FDR) test.

## Results

### Baseline Characteristics of Participants

Thirty-nine EC patients and 51 healthy controls were employed in this study. Baseline characteristics are shown in Table 1. There was no significant difference in sex. Differences in fiber, salt, fat, starch, meat, and vegetables/fruit consumption between the two groups were not significant either. There was no difference in smoking and drinking between the two groups, which seemed inconsistent with previous studies (Schueller et al., 2017; Fan et al., 2018; Hsiao et al., 2018). Average age of the EC patients was higher than that of the healthy controls. It may be caused by that the average age of EC diagnosis is higher. The EC patients took in less sugar than the healthy controls. No volunteer quit, and all samples met the analysis criterion.

### Microbiota Diversity of Saliva Samples of Esophageal Cancer Patients and Healthy Controls

**Figure 2:**
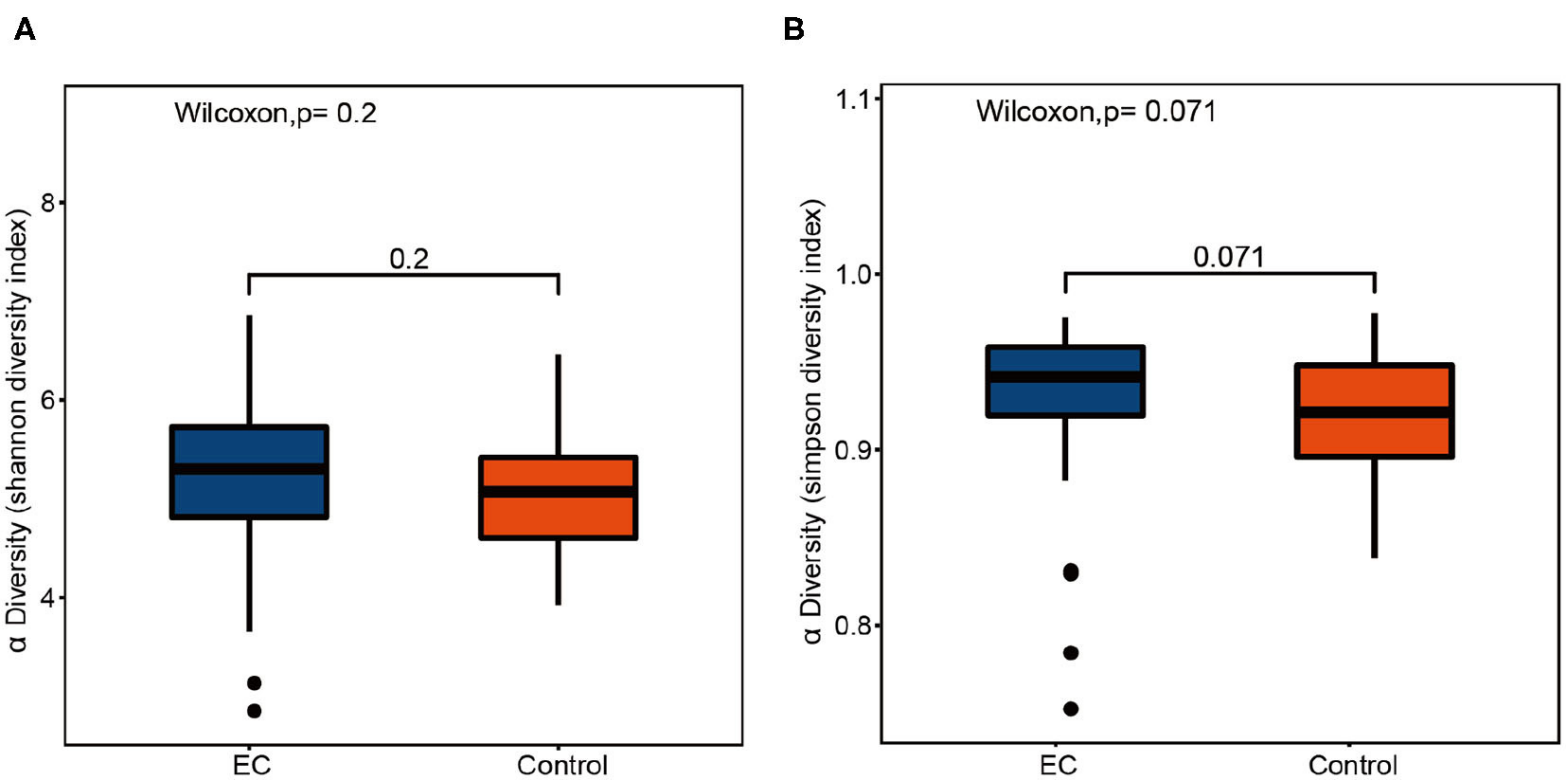
Box plots of alpha diversity indices comparing healthy controls' and esophageal cancer (EC) patients' saliva samples. The alpha diversity of oral microbiota shows no significant difference between the healthy controls and the EC patients. **(A)** Shannon diversity index, *p* = 0.2. **(B)** Simpson diversity index, *p* = 0.071.

**Figure 3:**
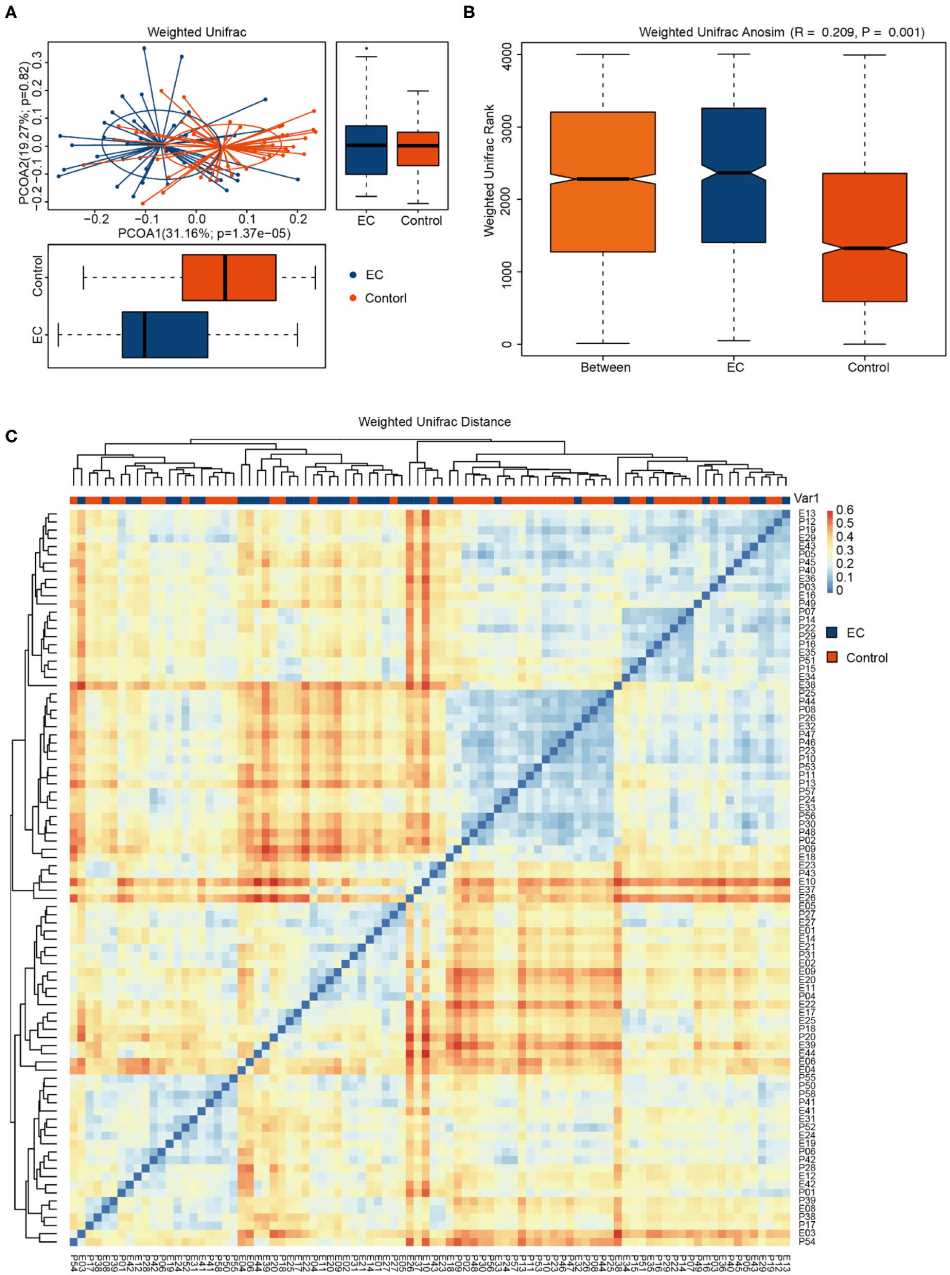
Comparisons of the phylogenetic structure and composition of saliva microbiota between esophageal cancer (EC) group and healthy group. **(A)** Principal coordinate analysis (PCoA) plot based on weighted UniFrac distance. The horizontal and vertical coordinates represent the first and second principal coordinates, respectively. Percentage represents the contribution rate of the corresponding principal coordinates to the difference of samples. P is the analysis *p*-value of the corresponding principal coordinates. Blue points represent the EC patients, while red points represent the healthy volunteers. Ellipses represent the 95% confidence interval (CI) around the cluster centroid. **(B)** Box plot of the Bray–Curtis dissimilarity distance-based analysis of similarities (ANOSIM) of the two groups. The abscissa represents all samples (Between) and each group, and the ordinate represents the rank of UniFrac distance. When *R* > 0, the difference between groups was greater than the difference within groups. When *R* < 0, the intragroup difference was greater than the intergroup difference. *p* < 0.05 indicates statistical significance. **(C)** Heatmap based on weighted UniFrac phylogenetic distances of the microbiota taxa among volunteers. The closer the samples are, the more similar the species composition of the two samples is.

Compared by Shannon diversity index (*p* = 0.2) and Simpson diversity index (*p* = 0.071), alpha diversity showed no significant difference between the EC group and the healthy group (Figure 2). There was no significant difference in microbial abundance and evenness between the two groups. PCoA results (Figure 3A) suggested that the composition and relative abundance of saliva microbiota of EC patients were different with healthy volunteers. A *p* = 0.001 of ANOSIM analysis based on Bray–Curtis dissimilarity distance supported the significance of difference (Figure 3B). Figure 3A showed similarities in the composition of the sample species and a trend of difference in species composition between the two groups. Heatmap results (Figure 3C) reflected similarities between the samples. By clustering the UniFrac results, we found similar beta diversities of the groups. It showed a trend of microbial diversity similarity among participants.

### Difference of Oral Microbial Taxa Between Esophageal Cancer Patients and Healthy Controls

**Figure 4:**
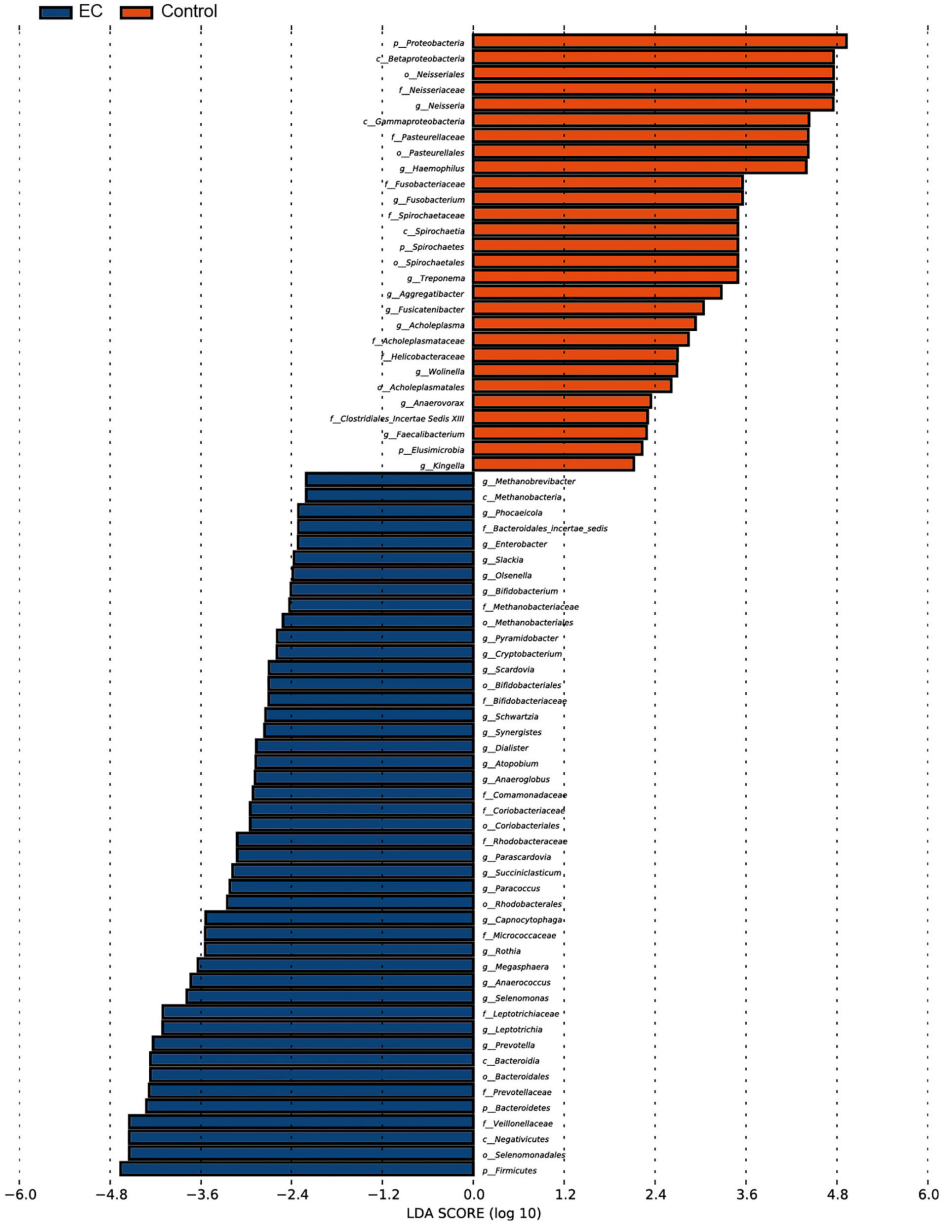
Microbial taxa difference between the esophageal cancer (EC) patients and the healthy controls. Significant enrichment of microbial taxa was identified in EC patients. Forty-six out of 74 taxa were identified to be more abundant in abundance of the EC patients than the healthy controls. Threshold linear discriminant analysis (LDA) score is 2, *p* < 0.05.

**Figure 5:**
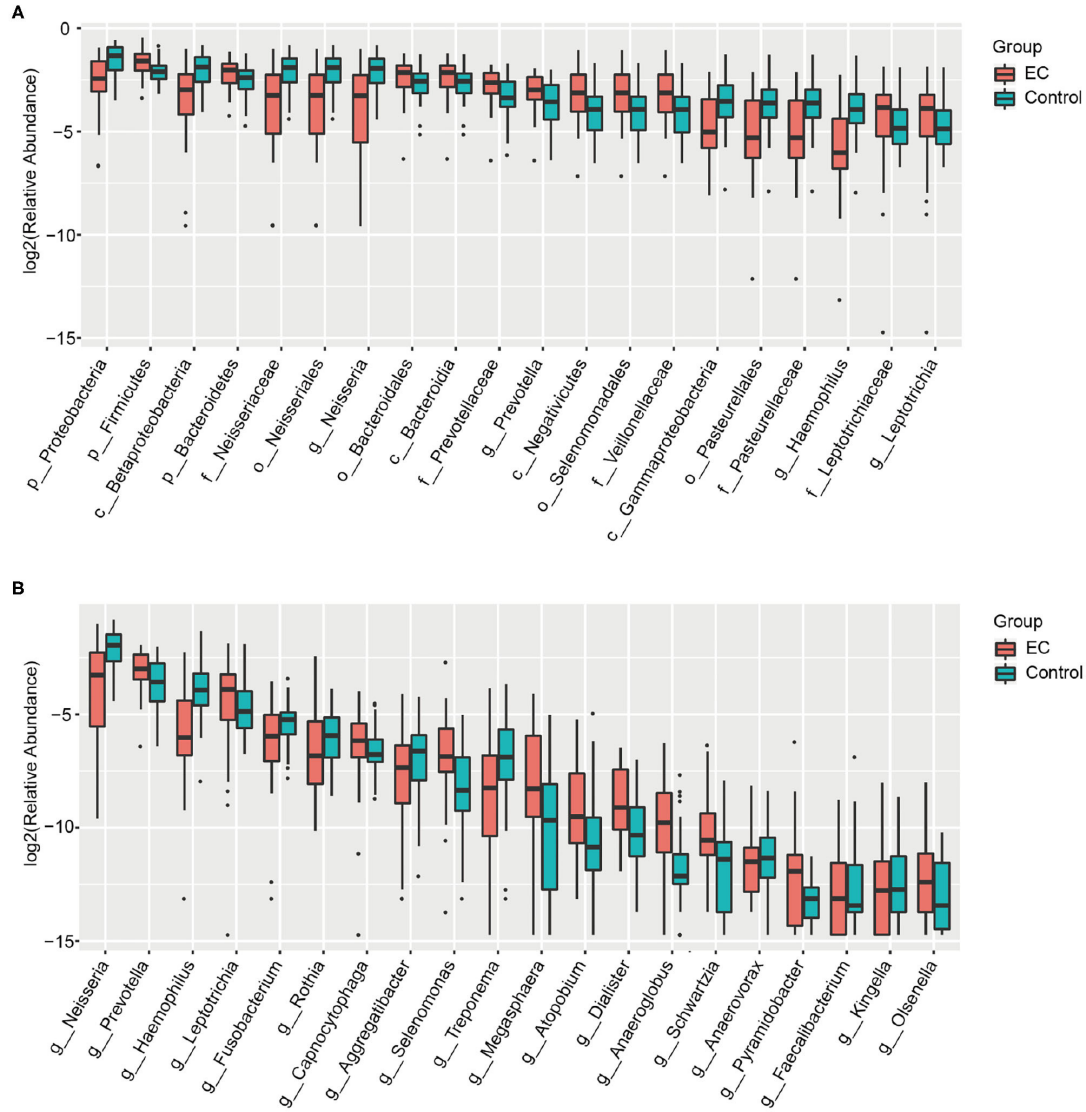
Box plots of the bacterial taxa differences between the healthy controls and the esophageal cancer (EC) patients. **(A)** Box plots of the top 20 different microbial taxa in abundance. **(B)** Box plots of the top 20 different microbial taxa in abundance at the general level.

We identified 1,298 microbial taxa from 90 samples, among which 1,250 were at the phylum level, 1,173 at the class level, 1,146 at the order level, 1,038 at the family level, and 667 at the genus level. Core microbiomes with 100% sample coverage and significant differences between groups were *Prevotella* and *Atopobium*. By using the LEfSe analysis with a log LDA score >2, we identified 74 taxa, of which 35 taxa were at the genus level, significantly different in abundance between the two groups (Figure 4). Then, we used Wilcoxon rank sum test (Wilcoxon test function of R stats package) to verify significant differences between the two groups in order to find out species that had significant differences in the division of groups. With a discriminant index of *p* < 0.05 and FDR *q* < 0.1, we identified 41 bacterial taxa significantly different in abundance between the two groups, of which 17 taxa were at the genus level (Table 2). For the EC patients, the most decreased floras were *Proteobacteria* at the phylum level (*p* < 0.001), *Betaproteobacteria* (*p* <0.001) at the class level, *Neisseriales* (*p* < 0.001) at the order level, *Neisseriaceae* (*p* < 0.001) at the family level, and *Neisseria* (*p* < 0.001) at the genus level, while the most enriched floras were *Firmicutes* (*p* = 0.006) at the phylum level, *Negativicutes* (*p* = 0.002) at the class level, *Selenomonadales* (*p* = 0.002) at the order level, *Veillonellaceae* (*p* = 0.002) at the family level, and *Prevotella* (*p* = 0.007) at the genus level. The top 20 microbial species with the largest different abundance are shown in Figure 5.

**Table 2:** Relative abundance of EC patients and healthy control.

**Taxon name**	**MAD (EC-C)**	***p*-values**	**FDR**
**Phylum**			
Firmicutes	9.53E-02	0.0006	0.0110
Proteobacteria	−1.77E-01	0.0000	0.0008
Spirochaetes	−5.70E-03	0.0011	0.0161
**Class**			
Betaproteobacteria	−1.22E-01	0.0000	0.0014
Gammaproteobacteria	−5.36E-02	0.0001	0.0039
Negativicutes	6.98E-02	0.0023	0.0297
Spirochaetia	−5.70E-03	0.0011	0.0161
**Order**			
Acholeplasmatales	−5.20E-05	0.0028	0.0299
Bifidobacteriales	8.98E-04	0.0000	0.0011
Coriobacteriales	2.19E-03	0.0003	0.0062
Neisseriales	−1.22E-01	0.0000	0.0013
Pasteurellales	−5.21E-02	0.0000	0.0014
Selenomonadales	6.98E-02	0.0023	0.0297
Spirochaetales	−5.70E-03	0.0011	0.0161
**Family**			
Acholeplasmataceae	−5.20E-05	0.0028	0.0299
Bifidobacteriaceae	8.98E-04	0.0000	0.0011
Clostridiales_Incertae Sedis XIII	−2.66E-04	0.0002	0.0041
Coriobacteriaceae	2.19E-03	0.0003	0.0062
Helicobacteraceae	−1.39E-04	0.0100	0.0930
Neisseriaceae	−1.22E-01	0.0000	0.0013
Pasteurellaceae	−5.21E-02	0.0000	0.0014
Prevotellaceae	4.23E-02	0.0062	0.0624
Spirochaetaceae	−5.70E-03	0.0011	0.0161
Veillonellaceae	6.98E-02	0.0023	0.0297
**Genus**			
*Acholeplasma*	−5.20E-05	0.0028	0.0299
*Anaeroglobus*	1.49E-03	0.0001	0.0029
*Anaerovorax*	−2.68E-04	0.0001	0.0031
*Atopobium*	1.81E-03	0.0010	0.0161
*Bifidobacterium*	2.75E-04	0.0025	0.0299
*Dialister*	1.57E-03	0.0027	0.0299
*Haemophilus*	−4.88E-02	0.0000	0.0008
*Megasphaera*	1.01E-02	0.0011	0.0161
*Neisseria*	−1.21E-01	0.0000	0.0013
*Olsenella*	2.08E-04	0.0036	0.0366
*Prevotella*	3.77E-02	0.0072	0.0704
*Pyramidobacter*	5.40E-04	0.0079	0.0750
*Scardovia*	3.97E-04	0.0028	0.0299
*Selenomonas*	1.21E-02	0.0004	0.0073
*Slackia*	1.43E-04	0.0109	0.0982
*Treponema*	−5.68E-03	0.0011	0.0161
*Wolinella*	−1.37E-04	0.0001	0.0035

*Only p < 0.05 and FDR q < 0.1 are shown. EC, esophageal cancer; MAD, mean abundance difference; FDR, false discovery rate*.

## Discussion

Our study supports that the oral microbiota in EC patients is different with healthy people. The abundance of *Prevotella* was enriched while *Neisseria* was decreased at the genus level in the EC patients, the change of which might be associated with an increased risk of EC.

A study assessing oral microbiome and its relationship with lifestyle, diet, hygiene, and socioeconomic and environmental parameters in 1,500 Spanish adolescents showed that microbiome samples could be divided into two broad grouping patterns (stomatotypes), driven by *Neisseria* and *Prevotella*, respectively (Willis et al., 2018). These stomatotypes might represent two possible optimal balances in the oral microbiome. The study suggested that the two stomatotypes might remain consistent across geographical regions, lifestyles, and ages. A similar study showed that esophageal microorganisms can also be divided into different community groups (esotypes), with *Streptococcus* and *Prevotella* as dominant bacteria (Deshpande et al., 2018). This study also proposed that age might be negatively correlated with abundance of *Prevotella* spp. of esophageal microbiome. Another study about healthy people suggested that esophageal microbiota were formed by colonization of oral floras and were similar to oral bacteria (Norder Grusell et al., 2013). In our study, the average age of EC patients was significantly older than that of healthy controls (*p* < 0.0001). Unlike healthy people mentioned above, the abundance of oral *Prevotella* in the older EC patients, compared with younger healthy controls, was significantly enriched. We propose that *Prevotella* may be associated with EC development. In addition, there was an age-matched case-control study about oral microbiota, and esophageal squamous cell carcinoma (ESCC) showed an increased relative abundance of *Prevotella* in ESCC patients (Chen et al., 2015). They also suggested the most abundant species were *Firmicutes* at the phylum level and *Prevotella* at the genus level, which was consistent with our results. *Prevotella* was also observed to be increased in patients with reflux esophagitis (RE) and BE (Liu et al., 2013). Several studies suggested that increased abundance of *Prevotella* in the oral cavity was associated with many diseases (Asakawa et al., 2018; Flemer et al., 2018; Liu et al., 2018; Wu et al., 2018; Yang et al., 2019), suggesting that it might be a promising biomarker for the occurrence of EC.

In our study, the increase of *Prevotella* abundance was only significant at the genus level and family level. It was one of core microbiomes that covered all samples. We believe the alteration of oral *Prevotella* may be a potential predictor of EC. However, we did not identify floras to the species level. A study showed that increased *Prevotella nanceiensis* was associated with a high risk of ESCC, while increased *Prevotella oral taxon 306* was associated with a low risk of ESCC, and increased *P. nanceiensis* was associated with a low risk of EAC (Peters et al., 2017). The relationship between *Prevotella* and EC needs further study.

The most abundance-increased bacteria from the phylum level to the genus level in our study all belonged to *Proteobacteria*, among which the most decreased was *Neisseria*. Peters et al. (2017) suggested that the decreased abundance of *Neisseria*, namely, *Neisseria sicca* and *Neisseria flavescens*, was associated with a low risk of EAC risk. However, only six patients of our study were diagnosed with EAC and the others were ESCC. A study about the relationship between oral flora and ESCC risk of Chinese patients also showed a decreased abundance of *Neisseria* in ESCC patients (Chen et al., 2015). Yet another study about the esophageal bacteria of patients with RE and BE in the US showed an increased tendency of *Neisseria* (Liu et al., 2013). It suggests further study about the composition of esophageal microbiota in EC patients. Contrary results about the *Nesseria* abundance with several studies of Europeans and Americans suggested the different oral microbiome composition of Chinese people, which might be influenced by diet and habits.

In addition, a study showed that decreased abundance of *Proteobacteria* and increased abundance of *Firmicutes* was associated with BE, which was consistent with our findings (Snider et al., 2018). We also observed *Veillonella*, a core microbiome, was significantly enriched in EC patients. It belongs to *Veillonellaceae, Selenomonadales, Negativicutes*, and *Firmicutes*, which were all the most enriched flora. Increased abundance of *Veillonella*, such as *Veillonella oral taxon 917*, was related to a high risk of EAC (Peters et al., 2017). A similar result was also observed to be increased in the gastroesophageal reflux disease (GERD) and BE patients' esophagus (Liu et al., 2013). Oral Gram-negative bacterial species was observed increased in the early stage of EAC patients, which supports our result (Deshpande et al., 2018).

Inspired by these studies, we will study the relationship between oral floras and serum tumor markers, inflammatory factors, and the differences of oral microbiota structures and functions in patients with EC at different stages. From which we may further understand the role of oral floras in the occurrence and development of EC and search for biomarkers for early prediction or prevention of EC and new targets for treatment of EC.

## Conclusion

Our study for the first time provides the diversity differences of oral microorganisms between EC patients and healthy people in China. Our study provides *Neisseria* and *Prevotella, Veillonella* as potential new biomarkers for EC. It also provides a direction for the study of the relationship between oral flora and EC.

## Data Availability Statement

The original contributions presented in the study are publicly available. This data can be found at: https://www.ncbi.nlm.nih.gov/sra/PRJNA660092.

## Author Contributions

QZ and BW participated in the design of this study and performed the statistical analysis. QZ collected the important background information, carried out the literature search, and drafted the manuscript. YZ and YY participated in the data analysis. BW and HC carried out the manuscript editing and review. All authors provided assistance for data acquisition, read, and approved the final manuscript.

## Conflict of Interest

The authors declare that the research was conducted in the absence of any commercial or financial relationships that could be construed as a potential conflict of interest.
